# A Nanoplex PCR Assay for the Simultaneous Detection of Vancomycin- and Linezolid-Resistant Genes in *Enterococcus*

**DOI:** 10.3390/diagnostics13040722

**Published:** 2023-02-14

**Authors:** Yusuf Wada, Azian Harun, Chan Yean Yean, Abdul Rahman Zaidah

**Affiliations:** 1 Department of Medical Microbiology and Parasitology, School of Medical Sciences, Universiti Sains Malaysia, Kubang Kerian 16150, Malaysia; 2 Department of Zoology, Faculty of Life Sciences, Ahmadu Bello University, Zaria 810211, Nigeria; 3 Hospital Universiti Sains Malaysia, Universiti Sains Malaysia, Kubang Kerian 16150, Malaysia

**Keywords:** *Enterococcus*, vancomycin, linezolid, multiplex PCR, resistant genes

## Abstract

Background: Enterococci are Gram-positive cocci found in the guts of humans and animals. The goal of this research is to develop a multiplex PCR assay that can detect the *Enterococcus* genus, four VRE genes, and three LZRE genes simultaneously. Methods: Primers used in this study were specifically designed for the detection of 16S rRNA of *Enterococcus* genus, *van*A—*van*B*—van*C*—van*D for vancomycin, *cfr* methyltransferase, and *optr*A*, and poxt*A, as well as an adenosine triphosphate-binding cassette (ABC) transporter for linezolid. A *Vibrio cholerae ctx*A (internal amplification control) was included. Optimization of primer concentrations and PCR components was also done. This was followed by evaluating the sensitivity and specificity of the optimized multiplex PCR. Results: Final Primer concentrations were optimized as follows: 16S rRNA is 1.0 pmol/μL, *van*A is 1.0 pmol/μL, *optr*A is 1.0 pmol/μL, *cfr* is 1.0 pmol/μL, *poxt*A is 0.1 pmol/μL, *van*B is 0.08 pmol/μL, *ctx*A is 0.07 pmol/μL, *van*C is 0.8 pmol/μL, and *van*D is 0.1 pmol/μL. Further, the optimized concentrations for MgCl_2,_ dNTPs and *Taq* DNA polymerase were 2.5 mM, 0.16 mM, and 0.75 units respectively, and an annealing temperature of 64.5 °C. Conclusions: The developed multiplex PCR is sensitive and species-specific. The development of a multiplex PCR assay that will take into account all known VRE genes and linezolid mutation is highly recommended.

## 1. Introduction

Enterococci, particularly *E. faecalis* and *E. faecium*, were once considered harmless commensals of the gastrointestinal tract; however, they are now playing a progressively critical role in hospital infections [1].

Vancomycin-Resistant Enterococcus (VRE) was reported in several countries around the world, and its prevalence is still on the rise. Vancomycin used to be the last treatment option for enterococcal infection until resistance began to emerge. Linezolid, a synthetic oxazolidinone group of antibiotics was designed to treat VRE. Being a synthetic antibiotic, resistance was not expected to be seen; however, linezolid-resistant *Enterococcus* (LZRE) and other bacteria species have been reported worldwide, although their prevalence is low. 

A global concern regarding development and health is antimicrobial resistance (AMR). To attain the Sustainable Development Goals, urgent multisectoral action is needed (SDGs) [2].

One of the top 10 worldwide public health hazards to humanity, according to the WHO, is AMR [2]. Drug-resistant infections are mostly brought on by the improper use and overuse of antibiotics. The proliferation of bacteria, some of which may be resistant to antibiotic therapy, is encouraged by a lack of clean water, proper sanitation, and effective infection prevention and control [2]. The economic burden of AMR is substantial. Long-term disease not only increases the risk of mortality and incapacity, but also lengthens hospital stays, necessitates the use of more expensive medications, and puts a strain on the finances of those affected [2]. Without efficient antimicrobials, infections would be more difficult to treat in modern medicine, notably during major surgery and cancer chemotherapy [3].

According to research published in The Lancet, 4.95 million people worldwide passed away in 2019 as a result of diseases in which bacterial AMR had a role. AMR was directly responsible for 1.27 million of those deaths, which means that drug-resistant bacteria killed more people than HIV/AIDS (864,000 deaths) or malaria (643,000 deaths) combined [4].

Thus far, no multiplex PCR (mPCR) has been developed for the simultaneous detection of both vancomycin- and linezolid-resistant genes in *Enterococcus*, making the one developed in this study the first, to the best of our knowledge. Yean et al. [5] developed a nanoplex PCR that simultaneously detects a bifunctional aminoglycoside- and vancomycin-resistant gene in *Enterococcus*. Further, Bender et al. [6] also developed a multiplex PCR for the detection of linezolid-resistant genes in *Enterococcus.*

The culture method is still regarded as the gold standard for the identification and detection of bacteria; however, the drawback is that the culture method often has low sensitivities, especially in clinical samples. Monoplex PCR, on the other hand, is laborious, especially when dealing with large samples and, consequently, they are not cost-effective and require multiple pipetting steps.

There is also a major worry that the genes that lead to vancomycin and linezolid resistance may be transmitted from enterococci to other bacteria, such as *Staphylococcus aureus*, for which vancomycin is one of the last-resort antibiotics, leaving few or no therapeutic interventions except for linezolid, to which it has also developed a resistance. 

Although using multiplex PCR to detect more than two targets simultaneously in a sample can save money and time, multiplex PCR is difficult to develop and is generally less sensitive than a monoplex PCR. Multiplex PCR has the benefit of being able to employ a set of primers as an internal amplification control. False negatives owing to botch reaction or false positives as a result of contamination are two potential issues with a basic PCR. Multiplex tests frequently indicate false negatives, as each amplicon serves as an internal check for other amplicons. Multiplex PCR uses less reagents and takes far less time to prepare than methods that require multiple tubes of monoplex PCRs [6,7,8]. A multiplex approach is best suited for sparing expensive polymerase and limited templates. In a multiplex PCR reaction, the quality of the template can be estimated more precisely than in a basic PCR reaction. Multiplex PCR’s rapid amplification and internal controls can be utilized to quantify the amount of a certain target in a sample. The quantity of standard template, reaction cycles, and the minimum restriction of the potential replication of product for each cycle must all be taken into account when using multiplex PCR to accurately quantify templates. 

Therefore, this study was designed to develop an mPCR assay for the simultaneous detection of the *Enterococcus* genus, an internal amplification control (IAC) gene, four VRE genes, and three LZRE genes.

## 2. Materials and Methods

### 2.1. Bacterial Reference Strains and Clinical Isolates

#### 2.1.1. Reference Strains

For this study, reference strains were obtained from ATCC, BCCM, and the Institute of Medical Research (IMR). These reference strains were utilized in the evaluation of monoplex and developed multiplex PCR assays. The list of the reference strains utilized in this study is shown in Table 1.

#### 2.1.2. Clinical Isolates

A total of 26 clinical isolates comprising 16 Gram-negative and 10 Gram-positive bacteria were utilized in this study for specificity evaluation. These isolates were obtained from the stock culture at the Medical Microbiology and Parasitology Department of Universiti Sains Malaysia, and are shown in Table 2.

#### 2.1.3. Synthetic dsDNA

Synthetic dsDNA utilized is the gBlocks™ (Integrated DNA Technologies, Coralville, IA, USA), which is a double-stranded gene fragment. Synthetic DNA were utilized in this study as positive amplification control (PAC) (*n* = 8) and internal amplification control (IAC) (*n* = 1). The characteristics of the synthetic DNA are shown in Table 3.

Synthetic dsDNA preparation was also carried out according to the manufacturer’s instructions (Integrated DNA Technologies, Coralville, IA, USA). Briefly, the tube was centrifuged for 3 min at 5000× *g*, ensuring that the DNA is at the bottom of the tube. Next, nuclease-free dH_2_O was added to achieve a 10 ng/μL concentration. The tube was then vortexed to ensure proper mixing and incubated at 50 °C for 20 min. Finally, it was briefly vortexed, centrifuged, and stored at −20 °C.

In preparing 100 μL of the synthetic dsDNA working solution with 1 ng/μL concentration, a 10 μL of synthetic dsDNA stock was suspended in 90 μL of nuclease-free dH_2_O and stored at −20 °C for further use.

### 2.2. Primers

Primers were designed to amplify the genes of interest. These genes were: 16S rRNA of *Enterococcus* genus, *van*A—*van*B—*van*C—*van*D for vancomycin, *cfr* methyltransferase, and *optr*A *and poxt*A; an adenosine triphosphate-binding cassette (ABC) transporter for linezolid. A *Vibrio cholerae ctx*A primer was adopted from the study of Yean et al. [5]. Primers for resistant genes were designed from the synthetic dsDNA utilized as the PAC. The clustalW program in Vector NTI version 9.0 software was used to design the primers. The primers were designed in such a way that their sizes were different from one another in the range of 50–150 bp (Table 4). The specificity of the designed primers was checked using the NCBI-BLAST program.

### 2.3. Preparation of DNA Templates from Clinical Isolates

The boiling procedure was used to prepare a bacterial cell lysate that included chromosomal and plasmid DNA for PCR amplification. In this approach, a colony of bacteria from an overnight agar plate was inserted in a 1.5 mL centrifuge tube with 50 μL distilled water. Following that, the bacteria suspension was boiled at 100 °C for 10 min after thorough mixing. After boiling, the tube was then centrifuged at 13,000× *g* (gravitational force). The supernatant was transferred into a sterile tube, either utilized right away for PCR amplification or kept for at least 4 weeks at 4 °C, and the pellets were discarded. 

### 2.4. Development of Nanoplex PCR

In this study, an internal amplification control (IAC) was incorporated in addition to utilizing a set of primers tailored for the simultaneous detection of vancomycin- and linezolid-resistant genes in *Enterococcus* in a multiplex PCR. These primers were then analyzed for their sensitivity utilizing the same species and specificity, utilizing different bacteria species that are Gram-positive and Gram-negative. A standard monoplex PCR was performed in a total volume of 20 μL containing 1 X PCR buffer (Apical Scientific Sdn.Bhd., Selangor, Malaysia), 2.5 mM magnesium chloride (MgCl_2_) (Apical Scientific Sdn.Bhd., Selangor, Malaysia), 0.2 mM deoxynucleotides (dNTPs) (Apical Scientific Sdn.Bhd., Selangor Malaysia), 1 μM of each sense and antisense primer, and 0.75 units of *Taq* DNA polymerase enzyme (Apical Scientific Sdn.Bhd., Selangor, Malaysia). 

The cycling conditions used in this study consisted of initial denaturation at 95 °C (5 min), 30 cycles of denaturation at 95 °C (30 s), annealing at 64 °C (30 s), elongation at 72 °C (30 s), and a final elongation at 72 °C (5 min). 

Then, to boost throughput and reliability, and reduce non-specific amplification, PCR components and conditions of the nanoplex were optimized based on the conditions of the monoplex PCR. Different concentrations of primer mixture ranging from 1.0 to 0.07 μM were prepared and tested on the PAC template. Following the primer optimization, optimization of dNTP concentration was done in the range of 0.08–0.24 mM. Different concentrations of MgCl_2_ were optimized in the range of 1.5–3.5 mM. Subsequently, *Taq* DNA polymerase enzyme was optimized in a range of 0.5–1.5 units. The IAC template concentration was optimized in the multiplex PCR in a range of 1 ng/μL–1 pg/μL. A set of T_a_ was utilized within 5 °C above and below the calculated T_a_ by using a gradient PCR thermal cycler. 

PCR products were separated by electrophoresis at 100 volts for 90 min on 2.0% agarose gel and stained with FloroSafe DNA stain (1ST BASE, Singapore Science Park II, Singapore). Lastly, the multiplex PCR was evaluated for its sensitivity and specificity after the optimization. 

## 3. Results

### 3.1. Primer Design and Analysis

**Figure 1:**
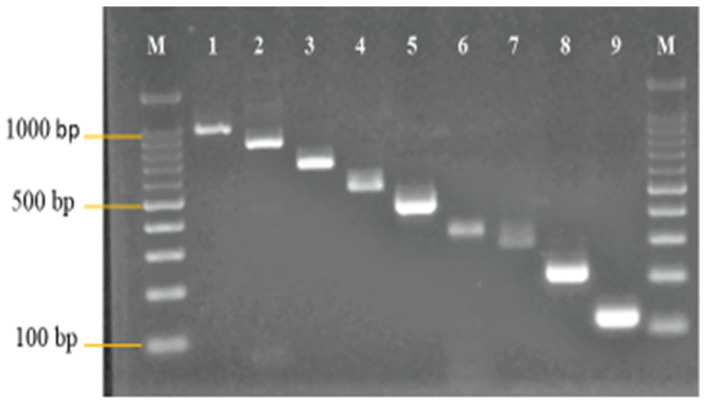
Monoplex PCR of 16S rRNA *Enterococcus*, *ctxA* (IAC), vancomycin and linezolid resistant genes primers. The differences in the sizes of the primers ranged from 50–150 bp. The designed primers were all amplified at their expected sizes. Lanes M: DNA ladder 100 bp plus. Lane 1: 16S rRNA *Enterococcus* (990 bp). Lane 2*: vanA* (850 bp). Lane 3: *optrA* (700 bp). Lane 4: *cfr* (550 bp). Lane 5: *poxtA* (450 bp). Lane 6*: vanB* (380 bp). Lane 7: *ctxA* (300 bp). Lane 8: *vanC* (220 bp). Lane 9: *vanD* (120 bp).

Primers were designed to amplify the genes of interest. These genes are: 16S rRNA of *Enterococcus* genus, *van*A—*van*B—*van*C—*van*D for vancomycin, *cfr* methyltransferase, and *optr*A *and poxt*A*, as well as* an adenosine triphosphate-binding cassette (ABC) transporter for linezolid. A *Vibrio cholerae ctx*A primer was adopted from the study of Yean et al. [5] (Figure 1). The primers for the resistant genes were designed from the synthetic dsDNA utilized as the PAC. The clustalW program in Vector NTI version 9.0 software was used to design the primers. The primers were designed in such a way that their sizes were different from one another in the range of 50–150 bp. The specificity of the designed primers was checked using the NCBI- BLAST program. 

Following the analysis of the designed primers in silico, in vitro analysis was also carried out. Standardized monoplex PCRs of the designed primers were performed to confirm the accuracy of the primers. Figure 1 shows the monoplex PCR of the designed primers at different sizes.

Subsequently, each of the primers were tested by evaluating their sensitivity to reference strains and specificity to the non-intended clinical isolates. All of the primers designed in this study were sensitive and specific. Synthetic dsDNA of the resistant genes was utilized as the positive control in the evaluation of the primers sensitivity and specificity.

### 3.2. Development and Optimization of the Nanoplex PCR

**Figure 2:**
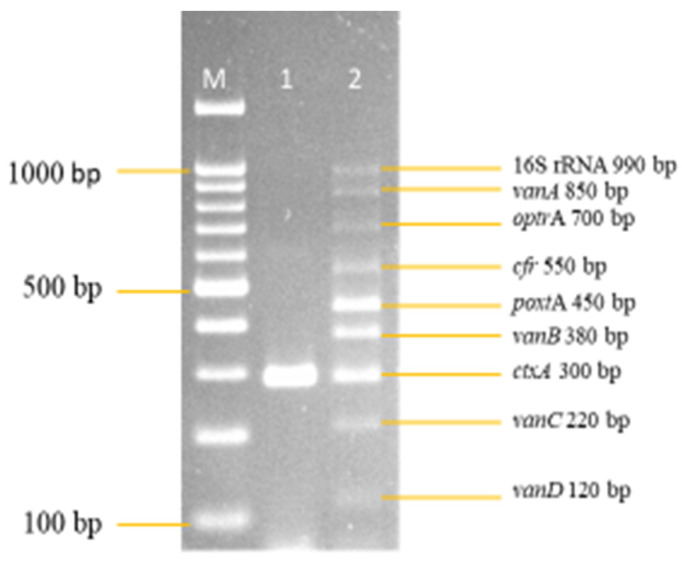
Final optimized multiplex PCR primer concentration. All other PCR parameters remained unchanged. Lanes M: DNA ladder 100 bp plus. Lane 1: Negative control with *ctxA* (Internal control). Lane 2: Optimized multiplex primer concentration. 16S rRNA = 1.0 pmol/μL. *vanA* = 1.0 pmol/μL. *optrA* = 1.0 pmol/μL. *cfr* = 1.0 pmol/μL. *poxtA* = 0.1 pmol/μL. *vanB* = 0.08 pmol/μL. *ctxA* = 0.07 pmol/μL. *vanC* = 0.8 pmol/μL. *vanD* = 0.1 pmol/μL.

Following the series of optimizations, all nine of the primer concentrations were finally optimized and are shown in Figure 2. The final primer concentration for 16S rRNA was 1.0 pmol/μL, *van*A was 1.0 pmol/μL, *optr*A was 1.0 pmol/μL, *cfr* was 1.0 pmol/μL, *poxt*A was 0.1 pmol/μL, *van*B was 0.08 pmol/μL, *ctx*A was 0.07 pmol/μL, *van*C was 0.8 pmol/μL, and *van*D was 0.1 pmol/μL.

A series of optimizations was carried out for the eight primers, including *ctx*A (IAC), to be amplified in the multiplex PCR assay. This occurred in a single-tube reaction and the optimal concentrations of the primers, MgCl_2_, dNTPs, and *Taq* DNA polymerase, were obtained. To amplify all nine genes simultaneously, the annealing temperature of the assay was also optimized. 

**Figure 3:**
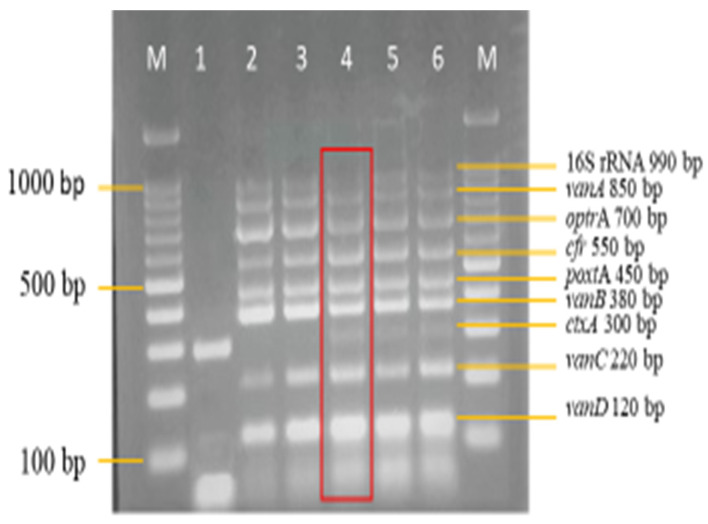
MgCl_2_ concentration optimization in the multiplex PCR. A concentration of 2.5 mM on lane 4 from a range of concentration (1.5–3.5 mM) was selected as the optimum concentration. Lane M: DNA ladder 100 bp plus. Lane 1: Negative control with *ctxA* (Internal control). Lane 2: 1.5 mM of MgCl_2_. Lane 3: 2.0 mM of MgCl_2_. Lane 4: 2.5 mM of MgCl_2_. Lane 5: 3.0 mM of MgCl_2_. Lane 6: 3.5 mM of MgCl_2_.

**Figure 4:**
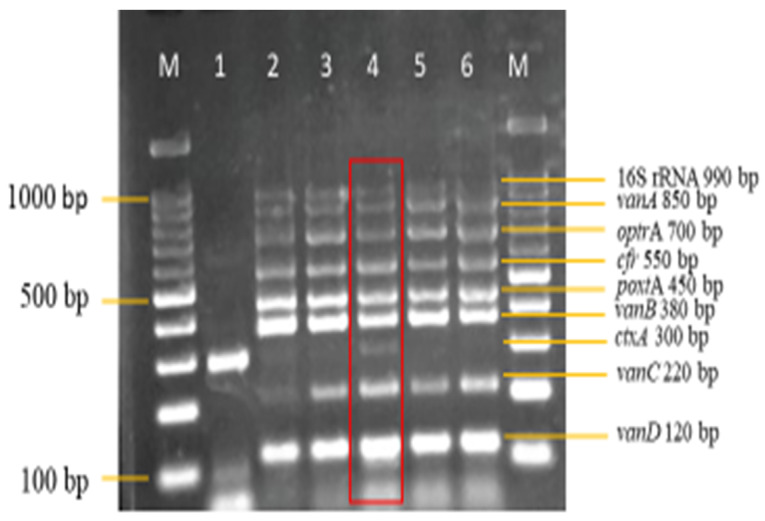
dNTPs concentration optimization in the multiplex PCR. A concentration of 0.16 mM on lane 4 from a range of concentrations (0.08–0.24 mM) was selected as the optimum concentration. Lane M: DNA ladder 100 bp plus. Lane 1: Negative control with *ctxA* (Internal control). Lane 2: 0.08 mM of dNTPs. Lane 3: 0.12 mM of dNTPs. Lane 4: 0.16 mM of dNTPs. Lane 5: 0.20 mM of dNTPs. Lane 6: 0.24 mM of dNTPs.

**Figure 5:**
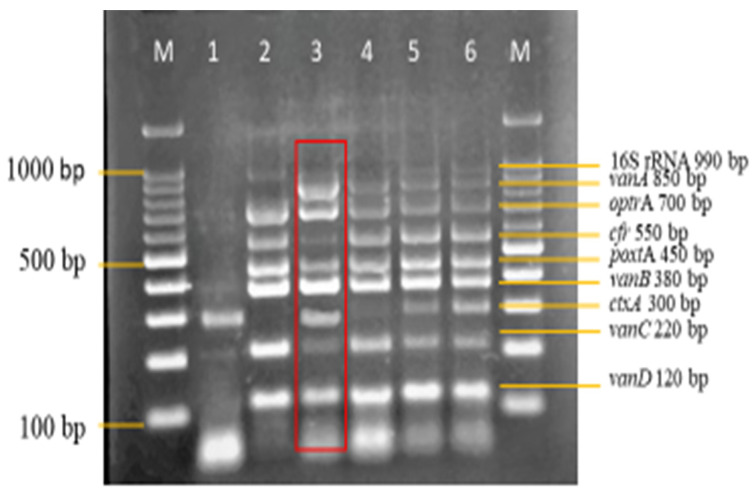
*Taq* DNA polymerase concentration optimization in the multiplex PCR. A concentration of 0.75 units on lane 3 from a range of concentrations (0.5–1.50 units) was selected as the optimum concentration. Lane M: DNA ladder 100 bp plus. Lane 1: Negative control with *ctxA* (Internal control). Lane 2: 0.50 Units. Lane 3: 0.75 Units. Lane 4: 1.00 Units. Lane 5: 1.25 Units. Lane 6: 1.50 Units.

**Figure 6:**
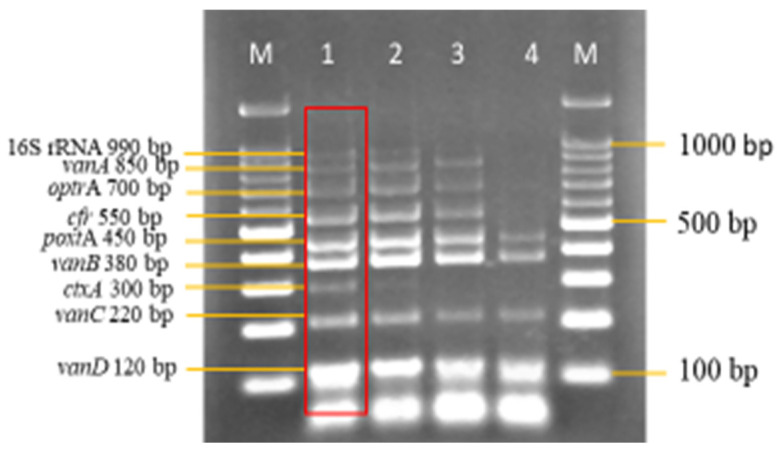
Synthetic dsDNA template concentration optimization in the multiplex PCR. A concentration of 1 ng/μL on lane 1 from a range of template concentrations (1 ng/μL–1 pg/μL) was selected as the optimum template concentration. Lane M: DNA ladder 100 bp plus. Lane 1: 1 ng/μL cocktail synthetic DNA template. Lane 2: 100 pg/μL cocktail synthetic DNA template. Lane 3: 10 pg/μL cocktail synthetic DNA template. Lane 4: 1 pg/μL cocktail synthetic DNA template.

**Figure 7:**
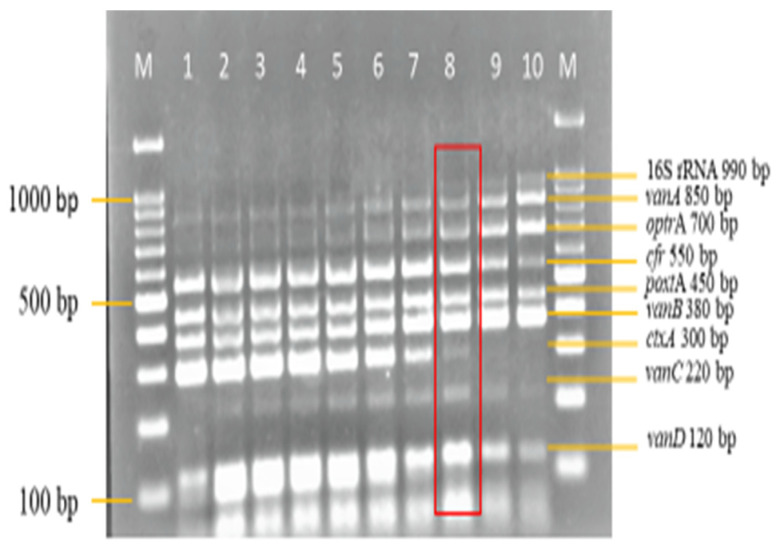
Annealing temperature optimization in the multiplex PCR. An annealing temperature of 64.5 °C on lane 8 from a range of temperature set (58–65.8 °C) was selected as the optimum annealing temperature. Lane M: DNA ladder 100 bp plus. Lane 1: 58 °C. Lane 2: 58.7 °C. Lane 3: 59.5 °C. Lane 4: 60.5 °C. Lane 5: 61.5 °C. Lane 6: 62.5 °C. Lane 7: 63.5 °C. Lane 8: 64.5 °C. Lane 9: 65.3 °C. Lane 10: 65.8 °C.

**Figure 8:**
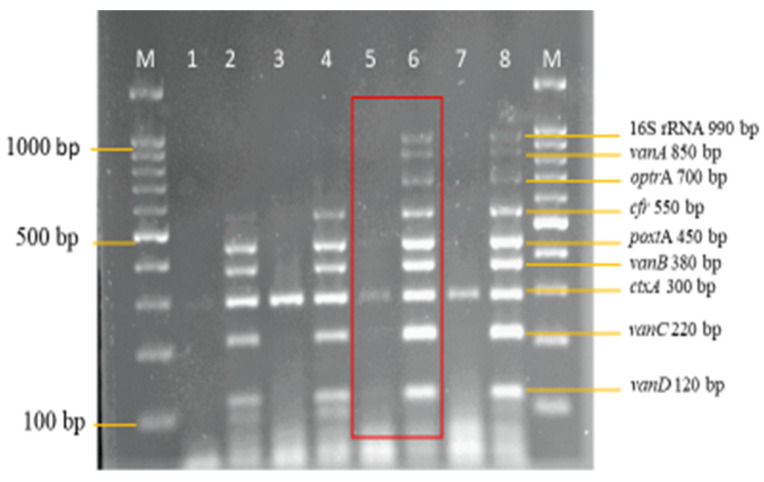
Internal amplification control template concentration optimization in the multiplex PCR Assay. A concentration of 10 pg/μL on lanes 5 and 6 from a range of template concentrations (1 ng/μL–1 pg/μL) was selected as the optimum template concentration. Lane M: DNA ladder 100 bp plus. Lane 1: 1 ng *ctxA* (Internal control). Lane 2: 1 ng *ctxA* (Internal control) with other targets. Lane 3: 100 pg *ctxA* (Internal control). Lane 4: 100 pg *ctxA* (Internal control) with other targets. Lane 5: 10 pg *ctxA* (Internal control). Lane 6: 10 pg *ctxA* (Internal control) with other targets. Lane 7: 1 pg *ctxA* (Internal control). Lane 8: 1 pg *ctxA* (Internal control) with other targets.

Therefore, 2.5 mM of MgCl_2_ on lane 3 was selected for further optimization (Figure 3). The optimum concentration of dNTPs selected in this study was 0.16 mM on lane 4 (Figure 4), as well, 0.75 units of *Taq* DNA polymerase on lane 3 was selected (Figure 5), synthetic dsDNA template concentration of 1 ng/μL on lane 1 was selected (Figure 6), the optimum annealing temperature selected for this study was 64.5 °C on lane 8 (Figure 7), and an IAC template concentration of 10 pg/μL was selected on lanes 5 and 6 (Figure 8).

#### Final Optimised Parameters of the Nanoplex PCR Assay

Following the series of optimizations performed in this multiplex PCR, the final optimized parameters of the multiplex PCR are outlined in Table 5.

### 3.3. Sensitivity and Specificity Evaluation of the Nanoplex PCR Assay

**Figure 9:**
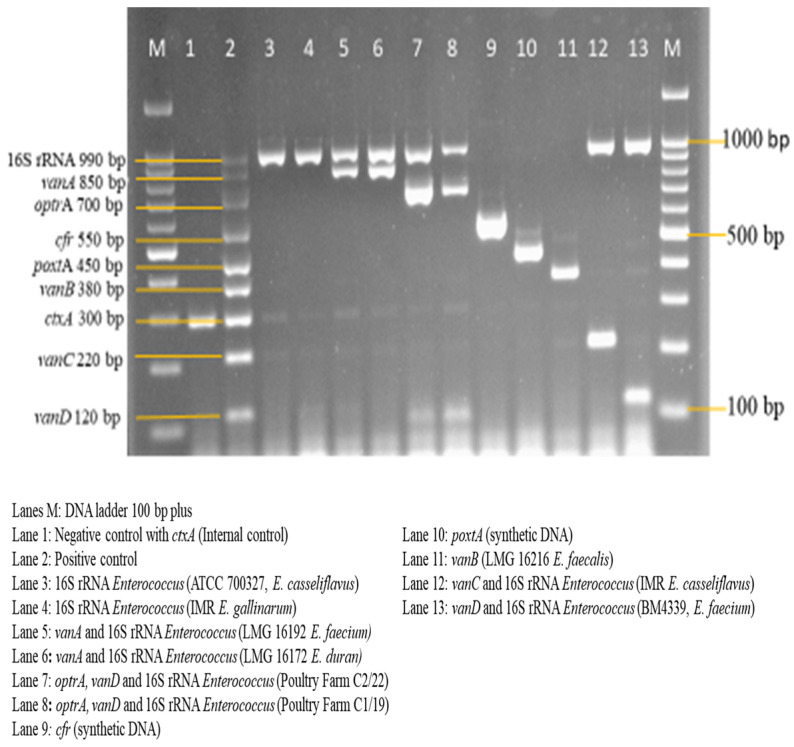
Sensitivity evaluation of multiplex PCR assay on intended reference bacterial strains (*n* = 11); 300 bp *ctx*A doubles as the negative control and IAC on lane 1 while lane 2, with all targets, was designated as the positive control.

**Figure 10:**
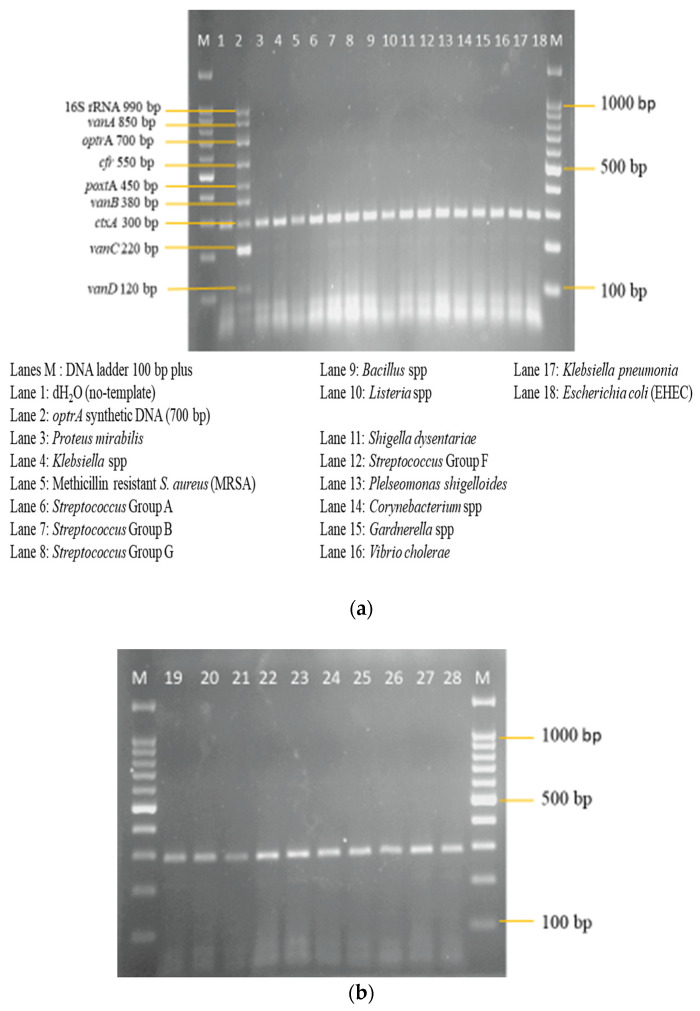
(**a**) Specificity evaluation of multiplex PCR assay on non-intended clinical isolates (*n* = 16). Amplification is absent except for the IAC, indicating the specificity of the multiplex PCR assay; 300 bp *ctx*A doubles as the negative control and IAC on lane 1, while lane 2, with all targets, was designated as the positive control. (**b**) Specificity evaluation of multiplex PCR assay on non-intended clinical isolates (*n* = 10) continued. Lane M: DNA ladder 100 bp plus. Lane 19: Escherichia coli (EPEC). Lane 20: *Vibrio parahaemolyticus*. Lane 21: *Shigella sonnei*. Lane 22: *Shigella boydii*. Lane 23: *Citrobacter freundii*. Lane 24: *Yersinia enterocolitica*. Lane 25: *Acinetobacter baumannii*. Lane 26: *Acinetobacter* spp. Lane 27: *Pseudomonas aeruginosa*. Lane 28: *Staphylococcus aureus*.

A sensitivity evaluation of the optimized multiplex PCR assay was carried out using 2 μL of extracted genomic DNA of the 11 target reference strains. These target strains included 550 bp *cfr* and 450 bp *poxt*A synthetic dsDNA. Synthetic dsDNA was utilized because of the unavailability of the *cfr* and *poxt*A reference strains. The result shown in Figure 9 shows the amplification of all 11 target reference strains, which were as expected. The optimized multiplex PCR assay was also evaluated for its specificity against 26 non-intended targets, which are clinical isolates. Here, also, 2 μL of DNA template of the isolates was utilized. The results in Figure 10a,b show the non-amplification of the non-intended targets with clear amplification of the IAC on all lanes.

## 4. Discussion

This study was designed to develop a nanoplex PCR assay to simultaneously detect vancomycin- and linezolid-resistant genes in *Enterococcus*. Vancomycin used to be the last treatment option for an enterococcal infection until resistance began to emerge. This resistance was thought to be a result of the overuse of the growth promoter Avoparcin in Europe, where the resistance was first seen. Subsequently, VRE has been reported in several countries around the world and its prevalence is still on the rise. Linezolid, a synthetic oxazolidinone group of antibiotics, was designed to treat VRE. Being synthetic antibiotics, resistance was not expected to be seen. However, linezolid resistance in *Enterococcus* and other bacteria species has been reported worldwide, although their prevalence is low. 

Thus far, no multiplex PCR has been developed for the simultaneous detection of both vancomycin- and linezolid-resistant genes in *Enterococcus*, meaning that this is the first, to the best of our knowledge. Yean et al. [5] developed a nanoplex PCR that simultaneously detect a bifunctional aminoglycoside and vancomycin-resistant genes in *Enterococcus*. Further, Bender et al. [6] also developed a multiplex PCR for the detection of linezolid-resistant genes in *Enterococcus.*

The multiplex PCR developed in this study includes four vancomycin-resistant genes (*van*A*, van*B, *van*C, and *van*D). These vancomycin-resistant genes were used in this study because they are the most commonly detected genes in clinical or environmental isolates. Similarly, three linezolid resistant genes (*cfr, optr*A, and *poxt*A) were also included in the multiplex PCR, as they are common and known resistant genes. This study is centered on *Enterococcus*; therefore, a 16S rRNA *Enterococcus* gene was included. The 16S rRNA *Enterococcus* gene is present in all *Enterococcus* species and highly conserved; therefore, this study did not take various *Enterococcus* species into consideration. Finally, a *ctx*A *Vibrio cholerae* gene was also included to function as the internal amplification control. This gene, from this bacterium, was used because it is Gram-negative, a non-target DNA, highly conserved, and not expected to amplify unless deliberately included in the reaction. This *ctx*A gene helps in ruling out the occurrence of PCR inhibitors and false-negative results in the multiplex PCR assay. In total, nine genes were included in the development of this multiplex PCR assay. 

The primer concentrations were optimized in this study. *Enterococcus* 16S rRNA, *van*A*, optr*A, and *cfr* all had a primer concentration of 1.0 pmol/μL. In the multiplex PCR of Yean et al. [5], an *Enterococcus* 16S rRNA primer concentration of 0.2 pmol/μL was reported, which was lower than that reported in this study, and a *vanA* primer concentration of 0.8 pmol/μL was also reported, which was closer to that reported in this study. Further, a primer concentration of 0.08 pmol/μL *van*B, 0.8 pmol/μL *van*C, 0.1 pmol/μL *van*D, and 0.07 pmol/μL *ctx*A was obtained in this study. This was also close to the primer concentration reported by Yean et al. [5] in their study. They reported a primer concentration of 0.05 pmol/μL *van*B, 0.7 pmol/μL *van*C, higher than that reported in this study, 0.4 pmol/μL *van*D closer to that reported in this study and 0.2 pmol/μL *ctx*A higher than that reported in this study. The *ctx*A *Vibrio cholerae* primer was adopted from the study of Yean et al. [5], and it is expected that their primer concentrations would be the same; however, they were not. The composition of other PCR parameters and the concentrations of different PCR primers could be responsible for this.

Similarly, the primer concentrations for linezolid-resistant genes were in contrast with those reported by Bender et al. [6] in their multiplex PCR. A primer concentration of 0.1 pmol/μL *poxt*A was reported in this study, which is the same *poxt*A primer concentration reported by Bender et al., 2019. However, Bender et al. [6] reported a primer concentration of 0.1 pmol/μL for both *optr*A and *cfr*, which varies from the primer concentration reported in this study. The differences in this primer concentration as observed in these studies could be a result of different concentrations of other PCR reagents and parameters. Further, the primers utilized in all of these studies were specifically designed to amplify their target genes and, as such, possess unique parameters, resulting in their varying concentrations in the multiplex PCR.

The optimization of MgCl_2_, dNTPs, and *Taq* DNA polymerase were also carried out. Magnesium chloride ions bind to the enzyme’s active site and increase its ability to perform the reaction. As a result, *Taq* DNA polymerase’s ability to add dNTPs to growing DNA strands is improved. Furthermore, MgCl_2_ enhances the reaction’s T_m_. Mg_2_^+^ ions in MgCl_2_ attach to the PO_3_^–^ and reduce the electrostatic barrier between DNA strands, temporarily protecting the negatively charged phosphate. The primer cannot connect to its exact site due to the electrostatic barrier between two DNA strands. The inclusion of MgCl_2_ facilitates the primer’s proper binding to its complementary bases. The right amount of MgCl_2_ improves the PCR specificity, whereas too much MgCl_2_ causes nonspecific binding, which reduces the accuracy and yield of the reaction. An MgCl_2_ concentration of 2.5 mM was observed in this study, which varies from the concentration (4.0 mM) reported by Yean et al. [5]. 

The function of deoxynucleotide triphosphates (dNTPs) in PCR is to help *Taq* DNA polymerase increase the growing DNA strand. They form hydrogen bonds with the complementary DNA strand. The amplification of all of the target genes in this multiplex PCR was dependent upon the successful optimization of the concentration of dNTPs. 

*Taq* DNA polymerase is derived from *Thermus aquaticus*, a bacterium that plays a thermophilic function in a PCR reaction to amplify the DNA in order to produce a myriad of DNA. It can also function at high temperatures because it is thermostable. It is useful in the last step of PCR, extension, in which *Taq* DNA polymerase synthesises the DNA region between the primers utilising dNTPs (denoxynucleoside triphosphates) and Mg_2_^+^. Subsequently, concentrations of 0.16 mM and 0.75 units were observed for dNTPs and *Taq* DNA polymerase, respectively, in this study, which also varies from those reported by Yean et al. [5] in their study (300 μM dNTPS and two units *Taq* DNA polymerase). Bender et al. [6] did not report concentrations for any of the above reagents, as their multiplex PCR optimization was done using a master mix. The annealing temperature, varying concentrations of primers, and other PCR reagent, could be accountable for the variances observed in the concentration of the aforementioned reagents. 

The importance of annealing temperature in a PCR cannot be overemphasized. An optimal annealing temperature is essential in a PCR because it determines the specificity of the PCR products. During the annealing phase of PCR, the reaction temperature must be low enough to allow both forward and reverse primers to bind to the template, but not low enough to allow the formation of unwanted, non-specific duplexes or intramolecular hairpins, both of which reduce reaction performance. The optimization of annealing temperature was carried out in a range of temperatures from 58 to 65.8 °C using a gradient program. In this study, an annealing temperature of 64.5 °C was utilized. However, this is close to the annealing temperature (65 °C) used by Yean et al. [5], and in contrast to that (50 °C) used by Bender et al. [6] in their multiplex PCR. An annealing temperature with a higher or lower degree could result in the impairment or inhibition of one or more of the target genes. The annealing temperature is dependent upon the composition of the nucleotide that makes up a primer. A primer with more guanine and cytosine nucleotide bases will most likely have a higher annealing temperature than a primer with adenine and thymine bases. This could be responsible for the variations observed in the annealing temperatures of these studies and, in addition, the concentration of other PCR reagents and parameters could also be responsible. 

The optimized nanoplex PCR assay was further tested for its sensitivity and specificity. Trevethan [10] defined sensitivity as a screening test’s ability to detect a true positive, while specificity is defined as a screening test’s ability to detect a true negative. A test was considered positive when all of the target amplicons were observed even though the IAC might be absent, while a negative test was affirmed negative only when an IAC was amplified. This is indicative that a test could be declared invalid when an IAC is not amplified in negative samples. Nik Zuraina et al. [11] developed a heptaplex PCR assay for the detection of six respiratory bacteria pathogens. Their developed heptaplex PCR, similar to our developed nanoplex, was sensitive and specific. Although the heptaplex PCR of Nik Zuraina et al. [11] was thermostabilised, it was able to detect true positive and true negatives samples.

Timely and accurate detection of vancomycin and linezolid resistant genes could enable appropriate treatment and minimize the spread of antibiotic resistance pathogens. This study utilized an end-point detection method for reading reaction of a PCR. Although this approach provides reliable results, it can be prone to cross-over contamination if not performed within a controlled environment. In addition, the nanoplex PCR assay which was developed in this study was not thermostabilised. A thermostabilised mPCR is known for its rapidity, simplicity, accuracy, and does not involve the use of cold-chain.

## 5. Conclusions

To the best of our knowledge, this is the first study to develop a multiplex PCR for the simultaneous detection of a 16S rRNA *Enterococcus* gene, four vancomycin-resistant genes, three linezolid resistant genes, and a *ctx*A *Vibrio cholerae* gene functioning as an IAC. This developed multiplex PCR is sensitive, species-specific, rapid, and capable of detecting vancomycin- and linezolid-resistant genes in all types of settings (clinical, environmental, and farm) because the resistant genes that were utilized have been reported in all of these settings. The *ctx*A IAC will also ensure that false-negative results and inhibitors are taken care of. The development of a multiplex PCR assay that will take into account all known VRE genes and linezolid mutations is highly recommended. This would ensure that no resistant genes are missed during routine laboratory diagnosis. Future research directions may include the development of a thermostabilised nanoplex PCR assay, taking into cognizance all known vancomycin- and linezolid-resistant genes.

## Figures and Tables

**Table 1:** Reference strains utilized for the sensitivity evaluation of multiplex and monoplex PCR assays.

Species	Reference	Target Gene
*Enterococcus casseliflavus* ^a^	ATCC 700327	16S rRNA *Enterococcus*
*Enterococcus gallinarum* ^b^	IMR	16S rRNA *Enterococcus*
*Enterococcus raffinosus* ^c^	LMG 12172	16S rRNA *Enterococcus*
*Enterococcus mundti* ^c^	LMG 12308	16S rRNA *Enterococcus*
*Enterococcus faecium* ^c^	LMG 16192	16S rRNA *Enterococcus*, *van*A
*Enterococcus durans* ^c^	LMG 16172	16S rRNA *Enterococcus*, *van*A,
*Enterococcus faecalis* ^c^	LMG 16216	*van*B
*Enterococcus casseliflavus* ^b^	IMR	16S rRNA *Enterococcus*, *van*C
*Enterococcus faecium* ^d^	BM4339	16S rRNA *Enterococcus*, *van*D
*Enterococcus casseliflavus* ^e^	Poultry Farm C2/22	16S rRNA *Enterococcus*, *optr*A, *poxt*A
*Enterococcus casseliflavus* ^e^	Poultry Farm C1/19	16S rRNA *Enterococcus*, *optr*A, *poxt*A

^a^ ATCC strain was obtained from the Department of Medical Microbiology and Parasitology, USM. ^b^ Obtained from the Institute of Medical Research, Malaysia. ^c^ Obtained from BCCM, Ghent Belgium. ^d^ Provided by Professor Patrice Courvalin and Dr Bruno Perichon, Institut Pasteur, Paris, France. ^e^ Obtained from Mohamad Nasir, N.S [9] and confirmed by sequencing.

**Table 2:** Clinical isolates utilized for evaluation of multiplex and monoplex PCR assays.

S/No	Bacteria Strains
	Gram-positive
1	Methicillin-resistant *Staphylococcus aureus* (MRSA)
2	*Streptococcus* Group A
3	*Streptococcus* Group B
4	*Streptococcus* Group G
5	*Streptococcus* Group F
6	*Bacillus* species
7	*Listeria* species
8	*Corynebacterium* species
9	*Staphylococcus aureus*
10	*Gardnerella* species
	Gram-negative
1	*Proteus mirabilis*
2	*Klebsiella species*
3	*Shigella dysentariae*
4	*Plesiomonas shigelloides*
5	*Vibrio cholerae*
6	*Klebsiella pneumoniae*
7	*Escherichia coli* (Enterohemorrhagic EHEC)
8	*Escherichia coli* (Enteropathogenic EPEC)
9	*Vibrio parahaemolyticus*
10	*Shigella sonnei*
11	*Shigella boydii*
12	*Citrobacter freundii*
13	*Yersinia enterocolitica*
14	*Acinetobacter baumannii*
15	*Acinetobacter species*
16	*Pseudomonas aeruginosa*

**Table 3:** Synthetic DNA utilized as positive and internal amplification control.

Synthetic dsDNA	Size (bp)
16S rRNA *Enterococcus*	993
*van*A	1032
*optr*A	880
*cfr*	725
*poxt*A	600
*van*B	1029
*ctx*A *	615
*van*C	699
*van*D	1032

* Internal Amplification Control.

**Table 4:** Characteristics of the primers used in this study.

Target Gene	Primer Sequence (5′-3′)	Product Length (bp)
16S rRNA *Enterococcus*	F-5′-TTC CAC CGG AGC TTG CTC C-3′ R-5′-TTT GCC CCC GAA GGG GAA G-3′	990
*van*A	F-5′-TTT GGG GGT TGC TCA GAG G-3′ R-5′-CAC ACG GGC TAG ACC TCT A-3′	850
*optr*A	F-5′-TGG AAA AAC AAC CTT GCT AAA AGC-3′ R-5′-CAA GCG TGT AAT CCT TTC AAT TTC-3′	700
*cfr*	F-5′-CAA AGA ATT AGT CGA TTT GAG GA-3′ R-5′-GTT CCT CAC TAT AAG GTG AGT-3′	550
*poxt*A	F-5′-TGC TTT TTC TCC AGG GGA CA-3′ R-5′-GTG GAG AGC TGC AAA AGA GA-3′	450
*van*B	F-5′-AAA ACG GCG TAT GGA AGC TAT G-3′ R-5′-CGG CTT CAC AAA GAC AGG GTA G-3′	380
*ctx*A *	F-5′-AAC TCA GAC GGG ATT TGT TAG GC-3′ R-5′-TCT CTG TAG CCC CTA TTA CGA TGT-3′	300 [5]
*van*C	F-5′-CAG CAG CCA TTG GCG TAC A-3′ R-5′-TGT AGG AGC ACT GCG GAA C-3′	220
*van*D	F-5′-AAG CTC CGT GAT CTG CAT GG-3′ R-5′-AAA TCC TCC GTT TCC AGG C-3′	120

*—Internal Amplification Control. F—Forward or Sense sequence. R—Reverse or Antisense sequence. bp—Base pair.

**Table 5:** Final optimized parameters of the nanoplex PCR assay for the detection of vancomycin- and linezolid-resistant genes in *Enterococcus*.

Components	Initial Concentration	Per Reaction (μL)	Final Concentration
PCR-Grade dH_2_O	-	2.37	-
10× Reaction Buffer	10×	2.0	1×
MgCl_2_	25 mM	2.0	2.5 mM
dNTPs	10 mM	0.32	0.16 mM
Primers (Sense and Anti-sense)			
16S rRNA *Enterococcus*	20 μM	1.0	1 μM
*van*A	20 μM	1.0	1 μM
*optr*A	20 μM	1.0	1 μM
*cfr*	20 μM	1.0	1 μM
*poxt*A	20 μM	0.1	0.1 μM
*van*B	20 μM	0.08	0.08 μM
*ctxA* (IAC)	20 μM	0.07	0.07 μM
*van*C	20 μM	0.8	0.8 μM
*van*D	20 μM	0.1	0.1 μM
*Taq* DNA Polymerase	5 units	0.15	0.75 units
*ctxA* Template (IAC)	10 ng	1.0	10 pg
Target DNA Cocktail mix (Synthetic dsDNA)	10 ng/μL of each target	2.0	1 ng/μL of each target
**Final Volume (μL)**		20	

## Data Availability

Dataset used and/or analyzed during the correct study are included in the manuscript.

## References

[1] Wada Y., Harun A.B., Yean C.Y., Zaidah A.R. (2019). Vancomycin-Resistant Enterococcus: Issues in Human Health, Animal Health, Resistant Mechanisms and the Malaysian Paradox. Adv. Anim. Vet. Sci..

[2] Antimicrobial Resistance. https://www.who.int/news-room/fact-sheets/detail/antimicrobial-resistance.

[3] Browne A.J., Chipeta M.G., Haines-Woodhouse G., Kumaran E.P.A., Hamadani B.H.K., Zaraa S., Henry N.J., Deshpande A., Reiner R.C., Day N.P.J. (2021). Global Antibiotic Consumption and Usage in Humans, 2000–2018: A Spatial Modelling Study. Lancet Planet. Health.

[4] Murray C.J., Ikuta K.S., Sharara F., Swetschinski L., Robles Aguilar G., Gray A., Han C., Bisignano C., Rao P., Wool E. (2022). Global Burden of Bacterial Antimicrobial Resistance in 2019: A Systematic Analysis. Lancet.

[5] Yean C.Y., Yin L.S., Lalitha P., Ravichandran M. (2007). A Nanoplex PCR Assay for the Rapid Detection of Vancomycin and Bifunctional Aminoglycoside Resistance Genes in Enterococcus Species. BMC Microbiol..

[6] Bender J.K., Fleige C., Klare I., Werner G. (2019). Development of a Multiplex-PCR to Simultaneously Detect Acquired Linezolid Resistance Genes Cfr, OptrA and PoxtA in Enterococci of Clinical Origin. J. Microbiol. Methods.

[7] Zangenberg G., Saiki R.K., Reynolds R. (1999). Multiplex PCR: Optimization Guidelines. PCR Applications.

[8] Song H., Bae Y., Jeon E., Kwon Y., Joh S. (2019). Multiplex PCR Analysis of Virulence Genes and Their Influence on Antibiotic Resistance in *Enterococcus* Spp. Isolated from Broiler Chicken. J. Vet. Sci..

[9] Mohamad Nasir N.S., Chan Y.Y., Harun A., Husin A., Kamaruzzaman N.F., Wada Y., Abdul-Rahman Z. (2021). Malaysian Journal of Microbiology Linezolid-resistant *Enterococcus casseliflavus* and *Enterococcus gallinarum* isolated from poultry farms in Kelantan, Malaysia. Malays. J. Microbiol..

[10] Trevethan R. (2017). Sensitivity, Specificity, and Predictive Values: Foundations, Pliabilities, and Pitfalls in Research and Practice. Front. Public Health.

[11] Nik Zuraina N.M.N., Goni M.D., Amalina K.N., Hasan H., Mohamad S., Suraiya S. (2021). Thermostable Heptaplex PCR Assay for the Detection of Six Respiratory Bacterial Pathogens. Diagnostics.

